# Bacteremia due to *Serratia rubidaea* in intensive care unit: a case series

**DOI:** 10.1186/s13256-023-04195-3

**Published:** 2023-11-19

**Authors:** Asma Mehdi, Ahlem Trifi, Salma Abbes, Eya Seghir, Bedis Tlili, Linda Masseoud, Azzouz Noussair, Asma Ouhibi, Hajer Battikh, Meriam Zribi, Sami Abdellatif

**Affiliations:** 1 Medical Intensive Care Unit, University Hospital Center La Rabta and Faculty of Medicine, University Tunis El Manar, Tunis, Tunisia; 2 Microbiology Department, University Hospital Center La Rabta and Faculty of Medicine, University Tunis El Manar, Tunis, Tunisia

**Keywords:** *Serratia rubidaea*, Bacteremia, Catheter infection, Thrombosis, Ketoacidosis, Intensive care, Cases report

## Abstract

**Introduction:**

Bacteremia caused by *Serratia rubidaea* is seldom mentioned in comparison with other Enterobacteriaceae species. It primarily affects immunocompromised patients undergoing invasive procedures. Furthermore, the incidence, clinical features, and microbiological profile of this pathogen in the intensive care unit are rarely described.

**Case presentation:**

We present four North African case studies of bacteremia in four young female patients admitted to the intensive care unit for ketoacidosis with a history of diabetes mellitus. All four patients developed catheter-related infections complicated by deep vein thrombosis. The catheter site was femoral in all cases, and the main clinical manifestation was poorly tolerated fever. The pathogen was isolated in multiple peripheral blood cultures (> 4) for each patient, showing a similar profile in all cases: resistance to third-generation cephalosporins and sensitivity to aminoglycosides, piperacillin, fluoroquinolones, and folate-pathway inhibitors. Targeted treatment consisted of a combination of ciprofloxacin 400 mg twice per day and trimethoprim/sulfamethoxazole 400/80 mg thrice per day for all four cases. However, in one case, this regimen was switched to amikacin due to adverse effects. The outcomes were favorable in the majority of cases. The patients described in this study were 21, 66, 22, and 27-year-old North African women.

**Conclusion:**

Most of the reported cases shared common risk factors and clinical aspects. Notably, a case of thrombosis complicating a catheter infection caused by *Serratia rubidaea* has not been previously reported in the literature. Furthermore, this bloodstream infection typically affects deeply immunocompromised patients. However, our four cases, admitted to the intensive care unit for ketoacidosis, only had a history of diabetes mellitus.

## Introduction

*Serratia rubidaea* is a Gram-negative bacterium belonging to the *Enterobacteriaceae* species. It is known as a zoonotic bacterium, primarily found in the environment, including water, soil, and vegetables [1]. Unlike other species of Enterobacteriaceae, *Serratia rubidaea* sporadically causes nosocomial infections [2, 3]. It predominantly affects immunocompromised patients undergoing invasive procedures or receiving prolonged broad-spectrum antibiotics [4]. While *Serratia. rubidaea* is commonly identified in clinical specimens from the respiratory tract, skin wounds, and bile, its detection in blood samples is rarely documented in the literature [5].

Furthermore, the incidence, clinical features, and microbiological profile of this pathogen in the intensive care unit (ICU) are infrequently reported. In light of this, we present four cases of *Serratia. rubidaea* bacteremia in critically ill patients, aiming to identify both commonalities and differences among them. This approach enhances our understanding of this rare infection.

Case reports

### Case 1

The first patient was a female 21-year-old North African Faculty of Sciences student with a history of diabetes mellitus and hyperthyroidism. Her usual medications were oral antidiabetic and antithyroid drugs. She was an active smoker. No family history was reported. She was admitted to the ICU for diabetic ketoacidosis and required intravenous insulin therapy via a left femoral catheter. At admission, oxygen saturation was 99% at ambient air, mean arterial pressure was 69 mmHg, heart rate was 62 beats per minute, body temperature was 37.5 °C, and Glasgow Coma Scale (GCS) was 15. Biological parameters at admission and during hospital stay are presented in Table 2. During her hospital stay, the patient presented an ST-elevated myocardial infarction. The coronary angiography showed no stenosis. Additional cardiac magnetic resonance imaging (MRI) revealed acute edematous myocarditis.

A total of 5 days after her admission, the patient presented a persistent fever at 40 °C with clinical intolerance: chills, tachycardia, and so on.

A computed tomography (CT) thorax, abdomen, pelvis (TAP) scan was performed revealing the presence of a left deep vein thrombosis extended to the common femoral vein, which explained the fever. C-reactive protein and procalcitonin levels were 306 mg/L and 36 ng/mL, respectively. We started a curative anticoagulation aligned with empiric broad-spectrum antibiotic therapy.

We conducted a bacteriological investigation, which revealed the presence of *Serratia rubidaea* in eight consecutive peripheral blood cultures. Simultaneously, the catheter tip culture identified the same pathogen with the same antimicrobial susceptibility (Table 1). Blood cultures for aerobic and anaerobic bacteria in addition to catheter tips were aseptically performed. Samples were quickly transported to the microbiological laboratory. The Gram stain showed Gram-negative coccobacilli. Blood samples were inoculated onto blood agar and MacConkey Agar (lactose nonfermenter), and incubated at 37 °C aerobically. After 24 hours of incubation, there was a pure growth of red-pigmented colonies. A quantitative technique was followed for colony count. A significant count (> 10^−3^ CFU/mL) was observed for the catheter’s tips. In addition, the ratio of central blood culture/peripheral blood culture was > 5. Further biochemical reactions showed catalase-positive, oxidase-negative, and beta-galactosidase-positive reactions. The bacteria did not ferment sorbitol and rhamnose but fermented glucose, mannitol, and inositol. Indole was not produced. An antimicrobial susceptibility test was performed on Mueller–Hinton agar using the disc diffusion test according to the recommendations of the Clinical and Laboratory Standards Institute (CLSI; 2020). The classes of antimicrobials tested were: beta lactams, aminoglycosides, quinolones (ciprofloxacin/levofloxacin), and sulfonamides. *Serratia. rubidaea* is intrinsically resistant to penicillin, first- and second-generation cephalosporins, macrolides, tetracycline, and colistin. The minimum inhibitory concentration of ciprofloxacin was not available.

**Table 1:** Susceptibility of *S. rubidaea* isolated in peripheral blood and catheter’s tips cultures for the four studied cases

Amoxicillin	Resistant
Amoxicillin-clavulanic acid	Resistant
Cefotaxime	Resistant
Ticarcillin	Sensitive
Piperacillin	Sensitive
Amikacin	Sensitive
Ciprofloxacin	Sensitive
Levofloxacin	Sensitive
Trimethoptrim-sulfamethoxazole	Sensitive

HBA1C: glycated haemoglobin; WBC: White Blood Cells ; LCT: Lymphocytes ; PH: Potential Hydrogen; HCO3-: bicarbonate ; BGL: Blood Glucose Level; AST: Aspartate transaminase; ALT: Alanine transaminase

The targeted treatment combined ciprofloxacin 400 mg twice per day and trimethoprim/sulfamethoxazole 400/80 mg thrice per day administered intravenously for 10 days. The duration of antibiotic treatment was guided by the progression of clinical and biological indicators. (Procalcitonin levels were 6 ng/mL on day 7 and 0.5 ng/mL on day 10.)

The patient was discharged 26 days after her admission. She was transferred to the Department of Infectious Diseases of our hospital. No physical or psychological sequelae were reported 6 months after her discharge.

### Case 2

A 66-year-old North African female patient with a medical history of hypertension, diabetes mellitus, chronic kidney disease, and dyslipidemia. Her regular medications included subcutaneous insulin, calcium channel blockers, and statins. She was a housewife and a mother of two healthy children, with no specific habits reported. Her admission to the ICU was due to diabetic ketoacidosis resulting from a urinary tract infection.

Upon admission, we initiated intravenous insulin therapy using a left femoral catheter and administered antibiotic treatment, including cefotaxime 6 g per day and ofloxacin 200 mg twice per day intravenously. Her initial vital signs at admission were as follows: oxygen saturation of 97% on ambient air, mean arterial pressure of 62 mmHg, heart rate of 67 beats per minute, body temperature of 38.5 °C, and a Glasgow Coma Scale (GCS) score of 15. Additional biological parameters at admission and during her hospital stay are presented in Table 2.

**Table 2:** Biological parameters throughout hospitalization for all patients

Biological parameters	WBC (× 10^3^/mm^3^)	Neutrophils (× 10^3^/mm^3^)	LCT (× 10^3^/mm^3^)	PH	HCO3- (mmol/L)	BGL (mmol/L)	Urea (mmol/L)	Creatinine (µmol/L)	AST (UI/L)	ALT (UI/L)	Lactate level (mmol/L)
Case 1: HBA1C level at admission: 13%											
Day 0:	25.75	21.6	2.32	7	6	18	4	67	16	14	1.4
Day 6:	6.730	5.99	0.6	7.30	18	29	2.62	84	20	16	1.7
At discharge:	9.620	6.95	1.85	7.49	22	6	5.74	43	9	11	0.9
Case 2: HBA1C level at admission: 10%											
Day 0	14.8	12.2	1.29	7.27	18.4	56	23	354	17	21	3
Day 6	11.3	7.29	2.73	7.44	18	6.39	6.26	96	24	14	1.9
Last day of hospital stay:	2.51	1.53	0.89	7	3	24	10.5	327	160	110	17
Case 3: HBA1C level at admission: 9.8%											
Day 0:	18.8	15.9	1.340	7.35	18.8	10.87	1.43	51.8	27	43	4
Day 6:	6.5	4.2	3.32	7.39	19.4	8.5	2.6	43	459	233	1.2
At discharge:	5.7	2.22	2.01	7.45	22.3	10	1.1	55	93	58	2.6
CASE 4: HBA1C level at admission: 8%											
Day 0:	12.47	11.72	0.54	7	5	20	3	88	10	6	3
Day 6:	8.93	7.11	1.047	7.41	22	12.8	2.38	57	14	9	1.4
Last day of hospital stay:	4.04	2.91	0.58	7.57	30	17.6	2.2	48	15	8	3.5

WBC, white blood cells; LCT, lymphocytes; BGL, blood glucose level; AST, aspartate transaminase; ALT, alanine transaminase

Concerning lower limb asymmetry, a Doppler echography revealed thrombosis in the external and internal iliac veins extending to the common left femoral vein. In this case as well, we initiated curative anticoagulation. Initially, there was an improvement in sepsis and ketoacidosis.

A total of 8 days after her ICU admission, the patient presented a persistent and poorly tolerated fever, reaching 41 °C. Further investigations revealed the presence of carbapenem-resistant *Klebsiella pneumoniae* in the catheter-tip culture. In response to the catheter infection, we escalated the antibiotic therapy to include imipinem and colistin. Subsequent investigations also identified *Serratia. rubidaea* in four consecutive peripheral blood cultures (see Table 1).

The data regarding bacteriological identification methods and susceptibility tests were similar to the first case.

The targeted treatment involved the intravenous administration of ciprofloxacin 400 mg twice per day and trimethoprim/sulfamethoxazole 400/80 mg thrice per day. However, after 72 hours of this combined therapy, we discontinued trimethoprim/sulfamethoxazole due to acute kidney injury, which required hemodialysis, as well as deep pancytopenia. Consequently, we switched to amikacin.

Unfortunately, the patient developed refractory septic shock with multisystem organ failure and passed away 21 days after her admission to the ICU.

### Case 3

A 22-year-old North African female patient, first-year Master’s degree student with a history of diabetes mellitus on subcutaneous insulin treatment. She neither smoked nor consumed alcohol, and there was no reported family history of medical conditions. Her admission to the ICU was due to diabetic ketoacidosis resulting from poor compliance with insulin therapy.

Upon admission, we initiated intravenous insulin therapy via a right femoral catheter. Her initial vital signs at admission were as follows: oxygen saturation of 98% on ambient air, mean arterial pressure of 70 mmHg, heart rate of 95 beats per minute, body temperature of 37 °C, and a Glasgow Coma Scale (GCS) score of 15. Additional biological parameters at admission and during her hospital stay are presented in Table 2.

A total of 3 days after her admission, the patient developed a poorly tolerated fever, reaching 40 °C, accompanied by chills and tachycardia. We conducted bacteriological sampling, including peripheral blood culture, urine, and endotracheal samples, and removed the catheter. Procalcitonin level was 14.13 ng/ml.

A CT-TAP scan revealed the presence of right deep vein thrombosis extended to the common femoral vein, along with a pulmonary embolism. Bacteriological investigations revealed the presence of *Serratia. rubidaea* in four peripheral blood cultures and the catheter tip culture (see Table 1). The methods for bacteriological identification and susceptibility tests were consistent with the previous cases.

The targeted treatment consisted of intravenous administration of ciprofloxacin 400 mg twice per day, trimethoprim/sulfamethoxazole 400/80 mg thrice per day, and curative anticoagulation, considering the presented catheter infection associated with deep thrombosis and bacteremia. Antibiotics were administered intravenously for 10 days. The duration of antibiotic treatment was guided by clinical and biological improvement, with the procalcitonin level dropping to 9 ng/mL on day 7 and 0.6 ng/mL on day 10.

After 3 days, due to the emergence of liver function disturbances (see Table 2), we discontinued trimethoprim/sulfamethoxazole. Subsequently, the liver function normalized.

The patient’s outcome was favorable, and she was discharged 11 days after admission and transferred to the infectious diseases department of our hospital. No physical or psychological sequelae were reported 6 months after her discharge.

### Case 4

A 27-year-old North African female patient with a history of diabetes mellitus and gastroduodenal ulcer was regularly taking subcutaneous insulin and proton pump inhibitors. She neither smoked nor consumed alcohol, and there was no reported family history of medical conditions. Her admission to the ICU was due to diabetic ketoacidosis triggered by a viral infection (tracheobronchitis). She had not received prior antibiotic therapy.

Upon admission, her vital signs were as follows: oxygen saturation of 98% on ambient air, mean arterial pressure of 67 mmHg, heart rate of 90 beats per minute, body temperature of 37 °C, and a Glasgow Coma Scale (GCS) score of 15. Additional biological parameters at admission and during her hospital stay are presented in Table 2.

The patient received insulin therapy via a femoral catheter in addition to standard treatment, and 3 days after admission, she developed a fever of 40 °C along with sepsis. A CT-TAP scan revealed bilateral extensive venous thrombosis from the femoral common vein to the primitive iliac vein. Simultaneously, curative anticoagulation was initiated along with empiric broad-spectrum antibiotics.

Bacterial investigations identified *Serratia rubidaea* in five peripheral blood cultures and the catheter tip culture (see Table 1). The methods for bacteriological identification and susceptibility tests were consistent with the previous cases.

The targeted treatment involved the intravenous administration of ciprofloxacin 400 mg twice per day and trimethoprim/sulfamethoxazole 400/80 mg thrice per day.

After 6 days, mechanical ventilation was instituted in response to respiratory failure. Pulmonary embolism was highly suspected because of the presence of signs of acute cor pulmonale on chest echocardiography.

Given the severe clinical presentation and a high procalcitonin level (increasing from 5 ng/mL to 16.5 ng/mL),we were unable to discontinue antibiotics.

Unfortunately, the patient suffered a sudden cardiac arrest and passed away 16 days after admission.

### Isolation and infection control

Upon the discovery of *Serratia rubidaea* within our ICU, a series of decisive actions were swiftly initiated to contain the outbreak and mitigate its impact. Foremost among these actions was the implementation of rigorous isolation and infection control measures. Infected or potentially infected patients were promptly isolated in designated areas. The ICU staff rigorously adhered to strict protocols. Continuous training was provided to ensure the meticulous donning and doffing of gowns, gloves, and masks when attending to infected patients. We emphasized the paramount importance of scrupulous hand hygiene and ensured that hand washing stations and hand sanitizer dispensers were made readily accessible throughout the unit. Furthermore, we directed our team to conduct frequent and thorough environmental disinfection procedures, with a special focus on high-touch surfaces using appropriate disinfectants. These infection control measures played a central role in containing the spread of *Serratia rubidaea* within our ICU environment.

### Source control

A comprehensive environmental assessment was conducted, encompassing a thorough examination of water sources, air handling systems, and medical equipment. Particular attention was given to water reservoirs and plumbing systems, as *Serratia rubidaea* is known to colonize water sources. Sampling and testing of water outlets, including taps and faucets, were performed to detect any presence of the pathogen.

## Discussion

This study presents four cases of bacteremia caused by *S. rubidaea*. All cases shared a catheter-related infection with deep vein thrombosis at the same femoral site. These patients were all admitted to the ICU due to diabetic ketoacidosis with a history of diabetes mellitus. All patients were female, and their primary clinical symptom was a poorly tolerated fever. The pathogen was consistently identified in multiple peripheral blood cultures (> 4) for each patient, suggesting a consistent profile among all four cases.

*Serratia rubidaea*, a red-pigmented, rod-shaped bacterium, was originally described by Stapp in 1940 as *Bacterium rubidaeum* and later reclassified as a *Serratia* species in 1973 [6]. While *S. marcescens* and *S. liquefaciens* are more commonly associated with infections linked to the *Serratia* genus, infections caused by *S. rubidaea* are relatively rare. It is an opportunistic pathogen that is seldom identified in humans [5].

In a study evaluating the Gram-negative microbiota of healthy equine oral cavities and their antimicrobial susceptibilities, two multidrug-resistant strains of *S. rubidaea* were found in two out of eight healthy horses. These strains were resistant to macrolides, aminoglycosides, and carbapenems [1]. Additionally, *S. rubidaea* was found in Italian food in 2018, where chicken würstel developed an unusual fuchsia pink color shortly after the package was opened, revealing the presence of *S. rubidaea* [7]. This pathogen is also found in soil and water, with the potential for transmission to humans [8].

While *S. rubidaea* is rarely identified in clinical specimens, its clinical significance cannot be entirely dismissed [8]. It is naturally resistant to penicillin G, oxacillin, cefazolin, cefuroxime, macrolides, lincosamides, streptogramins, glycopeptides, fusidic acid, and rifampicin. However, it is naturally sensitive to several aminoglycosides, piperacillin, piperacillin/tazobactam, carbapenems, some cephalosporins, fluoroquinolones, and folate-pathway inhibitors [9]. This pathogen has developed various mechanisms of antibiotic resistance, including multidrug efflux systems affecting quinolones and enzymatic systems impacting beta-lactams [9]. The strain found in our four cases showed sensitivity to quinolones, aminoglycosides, and folate-pathway inhibitors.

*Serratia rubidaea* primarily targets immunocompromised patients undergoing surgeries or other invasive procedures [10]. It is particularly likely to infect patients receiving broad-spectrum antibiotics [11]. In one instance, it was isolated from the bile and blood of a female patient undergoing surgery for bile tract carcinoma, with the infection resolving after treatment with ceftriaxone (1 g/12 hours) and tobramycin (100 mg /8 hours) [12].

Another case involved a 44-year-old woman treated for abdominal wall cellulitis who received intravenous daptomycin via a midline catheter. A total of 5 days later, she developed a fever, and investigations revealed the presence of *S. rubidaea* in the blood culture. Removal of the midline catheter and treatment with intravenous ceftazidime led to a successful outcome [13].

The shared characteristics between prior literature and our case studies were female gender and invasive medical procedures.

Additionally, Okada *et al*. documented a case involving a 48-year-old male patient with a history of alcoholic pancreatitis and diabetes mellitus. He was hospitalized due to symptoms of chills and vomiting, presenting with a body temperature of 38 °C, altered consciousness, tachypnea, tachycardia, and hepatomegaly. Laboratory analyses revealed a pronounced inflammatory response and hepatorenal dysfunction. Abdominal computed tomography unveiled the presence of multiple liver abscesses concomitant with portal vein thrombosis. The blood culture yielded *S. rubidaea*. Treatment with sulbactam/cefoperazone and tobramycin yielded favorable outcomes [14]. This study shares common features with our four cases, including the presence of diabetes mellitus, clinical intolerance to fever, and the occurrence of deep vein thrombosis (portal vein, in this particular case).

Mossad *et al*. were the first to report a case of *S. liquefaciens* catheter-related infection accompanied by deep thrombosis. Their report featured a 55-year-old male patient who had developed short gut syndrome as a complication of chronic pancreatitis and had been reliant on parenteral nutrition for the previous 6 months. The patient presented with neck pain along the tunnel of the indwelling intravenous central catheter and persistent fever. Four blood cultures confirmed the presence of *S. liquefaciens*. Ultrasound examination revealed an occlusive clot in the right internal jugular vein surrounding the central venous catheter. Echocardiography confirmed the existence of a mobile thrombus originating in the superior vena cava [15].

In contrast to the norm, we present the case of an immunocompetent patient exhibiting thoracic empyema due to *S. rubidaea*, which was identified in the pleural fluid [16].

## Conclusion

The majority of reported cases shared common risk factors, including admission to the ICU for diabetic ketoacidosis with a history of poorly controlled diabetes mellitus. The primary clinical manifestation across all cases was a poorly tolerated fever, and femoral venous catheters were prevalent, often complicated by vein thrombosis.

A notable finding from our study was the consistent occurrence of deep vein thrombosis in all catheter-related infections caused by *S. rubidaea*. To the best of our knowledge, no prior reports have documented thrombosis as a complication of catheter-related *S. rubidaea* infections.

Moreover, while *S. rubidaea* bloodstream infections typically target immunocompromised patients, our four cases admitted to the ICU for ketoacidosis had only a history of diabetes mellitus, indicating a unique presentation of this pathogen in relatively immunocompetent individuals.

These findings emphasize the importance of considering *S. rubidaea* as a potential pathogen in cases of catheter-related infections with unexplained fever, particularly in patients with diabetes mellitus, and underline the significance of addressing the associated risk of deep vein thrombosis in such cases. Further research is needed to better understand the clinical implications and management of these infections.

## Data Availability

All are available.

## Abbreviations

ICU: Intensive care unit
bid: Bis in die (twice per day)
tid: Ter in die (thrice per day)
